# Development and Validation of a New Clinical Scale for Infants with Acute Respiratory Infection: The ReSVinet Scale

**DOI:** 10.1371/journal.pone.0157665

**Published:** 2016-06-21

**Authors:** Antonio José Justicia-Grande, Jacobo Pardo-Seco, Miriam Cebey-López, Lucía Vilanova-Trillo, Alberto Gómez-Carballa, Irene Rivero-Calle, María Puente-Puig, Carmen Curros-Novo, José Gómez-Rial, Antonio Salas, José María Martinón-Sánchez, Lorenzo Redondo-Collazo, Carmen Rodríguez-Tenreiro, Federico Martinón-Torres, Respiratory Syncytial Virus network (ReSVinet)

**Affiliations:** 1 Translational Pediatrics and Infectious Diseases Section, Pediatrics Department, Hospital Clínico Universitario de Santiago de Compostela, Santiago de Compostela, Spain; 2 Genetics, Vaccines, Infections, and Pediatrics Research Group (GENVIP), Healthcare Research Institute of Santiago de Compostela, Santiago de Compostela, Spain; 3 Unidade de Xenética, Departamento de Anatomía Patolóxica e Ciencias Forenses, and Instituto de Ciencias Forenses, Grupo de Medicina Xenómica (GMX), Facultade de Medicina, Universidade de Santiago de Compostela, Galicia, Spain; Fondazione IRCCS Ca' Granda Ospedale Maggiore Policlinico, Università degli Studi di Milano, ITALY

## Abstract

**Background and Aims:**

A properly validated scoring system allowing objective categorization of infants with acute respiratory infections (ARIs), avoiding the need for in-person assessment and that could also be used by non-health professionals is currently not available. We aimed to develop a new clinical assessment scale meeting these specifications.

**Methods:**

We designed a clinical scale (ReSVinet scale) based on seven parameters (feeding intolerance, medical intervention, respiratory difficulty, respiratory frequency, apnoea, general condition, fever) that were assigned different values (from 0 to 3) for a total of 20 points.170 children under two years of age with ARI were assessed independently by three pediatricians using this scale. Parents also evaluated their offspring with an adapted version of the scale in a subset of 61 cases. The scale was tested for internal consistency (Cronbach’s alpha), Pearson correlation coefficient for the items in the scale, inter-observer reliability (kappa index) and floor-ceiling effect.

**Results:**

Internal consistency was good for all the observers, with the lowest Cronbach’s alpha being 0.72. There was a strong correlation between the investigators (r-value ranged 0.76–0.83) and also between the results obtained by the parents and the investigators(r = 0.73). Light’s kappa for the observations of the three investigators was 0.74. Weighted kappa in the group evaluated by the parents was 0.73. The final score was correlated with length of hospital stay, PICU admission and Wood-Downes Score.

**Conclusions:**

The ReSVinet scale may be useful and reliable in the evaluation of infants with ARI, particularly acute bronchiolitis, even with data obtained from medical records and when employed by parents. Although further studies are necessary, ReSVinet scale already complies with more score validation criteria than the vast majority of the alternatives currently available and used in the clinical practice.

## Background and Aims

Acute Respiratory Infections (ARI), and more specifically bronchiolitis, constitute a major cause of morbidity and mortality in infants throughout the world [1,2,3]. Even though the clinical characteristics, pathophysiology and course of ARI in infancy are well known, there is no consensus with regards to how best to measure the severity of disease and the impact of therapeutic or preventive interventions in clinical trials [4, 5].

Many clinical scores have been published and even validated for the evaluation of infants with ARI and/or bronchiolitis, although none of them has been sufficiently validated to allow meaningful use in children [5]. These scales incorporate a variety of end points, and they all have important limitations. These include the following: they are designed for medical personnel, they rely on in-person assessment of the patient by a physician (neglecting clinical records and parental opinion), many of them are focused only in the respiratory status of the patient, and in most cases they require parameters that are not universally available (such as oxygen saturation or invasive procedures) [6,7].

No score has been validated as a research tool in the setting of pediatric respiratory infections; many of them are focused only in the respiratory status of the patient, and most, if not all, require in-person evaluation, which may be particularly difficult to ensure in outpatient clinical trials [5, 7]. We also currently lack a scale that could be used by parents and physicians alike. Clinicians in emergency departments and primary settings, as well as professionals involved in outpatient clinical trials, could all benefit greatly from such a scoring system. Doctors could have a reliable tool, allowing parents to monitor the evolution of children at home, establishing objective thresholds for patient referral or to seek of medical assistance. In addition, in the setting of clinical trials, having a validated score that parents can use would allow objective follow-up after hospital discharge or in case of mild diseases.

The goal of this project was to develop and evaluate a global clinical severity scale (ReSVinet scale) that would be amenable to be used in outpatient and inpatient studies without the need of in-person assessment. Internal reliability, construct validity, and ease of applicability of the scale were assessed. Additionally, the validity and reproducibility of an adapted version of this score in non-medical language for parental use was evaluated.

## Methods

All necessary ethical permissions were obtained from the regional ethics committee in Galicia (Spain) prior to the beginning of this study, which was approved by the aforementioned Institution on 23 March 2010 (registration number 2010/015). Written informed consent was obtained from all participants’ legal guardians before each patient was included in the study.

### a- Score design

The main investigators, with substantial research experience in pediatric respiratory infections, systematically reviewed studies that reported on the use of a respiratory score in children with ARI. The following symptoms and clinical findings were initially considered as indicative of disease severity: general condition, respiratory difficulty, oxygen saturation by pulse oxymeter, cyanosis, apnea, food tolerance, tachypnea, and tachycardia. Finally some of these symptoms were either included as part of a single component of the score, or excluded for being considered redundant or for limiting the applicability of the score in non-hospital settings and/or by non-medical personnel.

The proposed score was submitted for evaluation to 90 pediatricians of outpatient, hospital care and pediatric critical setting from the Galician Pediatric Research Network (REGALIP) and from the FIVE research group [8]. They agreed on the ease of recording and value of the parameters included. The final ReSVinet scale is detailed in **Table 1**, consisting of seven different items with different value ranges assigned, which add up to a total score that spans from 0 to 20.

**Table 1:** ReSVinet scale. This table presents the original scale, and was the one used by the three investigators.

	Item	0 points	1 points	2 points	3 points
**1**	**Feeding intolerance**	**No**	**Mild** Decreased appetite and/or isolated vomits with cough.	**Partial** Frequent vomits with cough, rejected feed but able to tolerate fluids sufficiently to ensure hydration.	**Total** Oral intolerance or absolute rejection of oral feed, not able to guarantee adequate hydration orally. Required nasogastric and/or intravenous fluids
2	**Medical intervention**	**No**	**Basic** Nasal secretions aspiration, physical examination, trial of nebulized bronchodilators, antipyretics.	**Intermediate** Oxygen therapy required. Complementary exams were needed (chest X-rays, blood gases, hematimetry…). Maintained nebulized therapy with bronchodilators.	**High** Required respiratory support with positive pressure (either non-invasive in CPAP, BiPAP or high-flow O2; or invasive through endotracheal tube).
3	**Respiratory difficulty**	**No**	**Mild** Not in basal situation but does not appear severe. Wheezing only audible with stethoscope, good air entrance. If modified Wood Downes, Wang score or any other respiratory distress score is applied, it indicates mild severity.	**Moderate** Makes some extra respiratory effort (intercostal and/or tracheosternal retraction). Presented expiratory wheezing audible even without stethoscope, and air entrance may be decreased in localized areas. If modified Wood Downes, Wang score or any other respiratory distress score is applied, it indicates moderate severity.	**Severe** Respiratory effort is obvious. Inspiratory and expiratory wheezing and/or clearly decreased air entry. If modified Wood Downes, Wang score or any other respiratory distress score is applied, it indicates high severity.
4	**Respiratory frequency**	**Normal** < 2 m: 40–50 bpm 2–6 m: 35–45 bpm 6-12m: 30–40 bpm 12-24m:25–35 bpm 24-36m: 20–30 bpm	**Mild or occasional tachypnea** Presented episodes of tachypnea, well tolerated, limited in time by self-resolution or response to secretion aspiration or nebulization.	**Prolonged or recurrent tachypnea** Tachypnea persisted or recurred despite secretion aspiration and/or nebulization with bronchodilators.	**Severe alteration** Severe and sustained tachypnea. Very superficial and quick breath rate. Normal/low breath rate with obvious increased respiratory effort and/or mental status affected. Orientative rates of severe tachypnea: < 2 m: > 70 bpm 2–6 m: > 60 bpm 6-12m: >55 bpm 12-24m: >50 bpm 24-36m: >40 bpm
5	**Apnea**	**No**			**Yes** At least one episode of respiratory pause medically documented or strongly suggested through anamnesis.
6	**General Condition**	**Normal**	**Mild** Not in basal situation, child was mildly uncomfortable but does not appear to be in a severe condition, not impress of severity. Parents are not alarmed. Could wait in the waiting room or even stay at home.	**Moderate** Patient looks ill, and will need medical exam and eventually further complementary exams and/or therapy. Parents are concerned. Cannot wait in the waiting room.	**Severe** Agitated, apathetic, lethargic. No need of medical training to realize severity. Parents are very concerned. Immediate medical evaluation and/or intervention were required.
7	**Fever**	**No**	**Yes, mild** Central T < 38.5°C	**Yes, moderate** Central T > 38.5°C	

(m = months)

We aimed to validate this scale using information obtained from patients’ medical records. Our secondary aim was to provide the parents with a simple tool for evaluating the severity of their children’s disease. The scale was thus developed to cover an important limitation of the scoring systems currently available: none of them was designed for use by non-medical personnel. All the components as explained in the scale should be easily evaluated by a parent/caregiver with no previous training. This resulted in an adapted version of the ReSVinet scale written in plain Spanish language (**Table 2**).

**Table 2:** Adapted version of the ReSVinet scale for parental use.

	Item	0 points	1 points	2 points	3 points
**1**	**Feeding intolerance**	**No**	**Mild** Decreased appetite (the child did not eat the same as normally) and/or presented isolated vomits with or without cough.	**Partial** Frequent vomits with cough, but the child does not vomit with every intake. Feeding exhausts the child.	**Total** Child is unable to feed him/herself. The use of a nasogastric tube or parenteral nutrition was required.
2	**Medical intervention**	**No**	**Basic** The child’s respiratory secretions required removal, he or she was explored by a physician or received sporadically nebulized medication. Antipyretics were administered.	**Intermediate**The child required oxygen therapy, underwent a chest X-ray exploration, or a blood sample was extracted. Treatment with nebulized drugs was given regularly.	**High** The child required respiratory support with a machine. Respiratory support was given through a special mask applied on the nose or mouth or resting on the child’s face, or through an endotracheal tube.
3	**Respiratory difficulty**	**No**	**Mild** The child was not breathing normally, but he/she does not seem to have any difficulty when drawing air.	**Moderate** The child made an effort for breathing. Respiratory noises can be heard without the need of a stethoscope (just approaching the ear to his or her chest).	**Severe** Respiratory effort was obvious. The child made important movement of his/her chest, the chest even collapses with every movement, and muscles of neck and belly were used. A lot of respiratory noise was heard without approaching the ear to the child’s chest.
4	**Respiratory frequency**	**Normal**	**Mild or occasional tachypnea** The child breathed more rapidly, but the situation was well tolerated, or the respiratory frequency was normalized after removing secretions from respiratory airways or administering nebulized medication.	**Prolonged or recurrent tachypnea** The child breathed more rapidly in a more persistent manner, even after receiving nebulized medication or removing secretions from respiratory tract.	**Severe alteration** The child breathed quickly and superficially, or really deeply. The child was agitated or drowsy. Orientative rates of severe tachypnea:
5	**Apnea**	**No**			**Yes** The child stopped breathing. It may have been necessary to stimulate him/her in order to regain normal breathing rate.
6	**General Condition**	**Normal**	**Mild** Child did not seem the same as always, but there did not seem to be anything to worry about.	**Moderate** Child looked ill, and medical examination was required, but it did not feel like a life-threatening situation.	**Severe** Child was agitated, apathetic,and/or lethargic. He/she required urgent medical attention. There was no need to be a doctor to see that the clinical situation of the child is worrying.
7	**Fever**	**No**	**Yes, mild** Rectal or tympanic temperature < 38.5°C, or axillar temperature < 38°C	**Yes, moderate** Rectal or tympanic temperature > 38.5°C, or axillar temperature > 38°C	

### b- Study Population

Patients were prospectively recruited among those admitted during two consecutive seasons in three hospitals in Spain. Although the ReSVinet scale was initially conceived as a tool for the entire pediatric age scope, a homogeneous group was defined in order to minimize biases: only previously healthy children under two years of age with the diagnosis upon admission of acute respiratory infection were included in the study. Infants with relevant underlying diseases (including cardiac disease, chronic pulmonary problems, anatomic malformations, neurologic diseases, severe malnutrition, immunodeficiency or hematologic malignancies) were excluded. The parents of those fulfilling the above criteria were asked to participate in the study right after their children had been admitted and, if informed consent was given, infants were allocated to one of the following two cohorts:

- In **group 1 (professional; G1)**, the clinical records of 170 children were retrospectively evaluated using the new scale by three physicians (Observer 1- **O1**-, Observer 2 –**O2**-, Observer 3 –**O3**-) with expertise in the emergency setting.
- In **group 2 (parental; G2)**, the clinical status of 61 patients was assessed by their caregivers at the moment of discharge using an adapted version of the scale. Their results were compared to those obtained by a physician blinded to the parental score (**O1**) who reviewed the medical history of patients from G2 upon discharge. When parents consented to take part in the study, simple directions on how to complete the scale were offered by a study nurse, who outlined the different items reading them to the parents to make sure that they were perfectly understood, and offered them the possibility of asking their doubts about the study. Parents should answer those questions using their own conception about severity in each item. They had no access to medical records. Respiratory rate references were avoided in the parental version as their inclusion could require standardized training. Whenever possible, infants were always fed by parents. Children were clinically assessed on a daily basis by a doctor not involved in the evaluation of the score. The same doctor then offered the legal tutors information on the evolution of the illness, treatment options and test to be performed as a part of routine care. Routine care was the same for all infants, whether they were taking part in the study or not. Physicians in charge of scoring the severity of the illness did not take part in the everyday clinical care of those children, and made no decisions on the tests a patient should undergo or on treatment changes. At the moment of discharge, the caregivers completed their version of the ReSVinet scale as if the child was in his or her worst moment during admission.

In both sets of patients, clinical information and demographic data were registered in standardized forms by either investigators or parents. A descriptive analysis of the recorded variables was performed.

### c- Score assessment

Our scale was tested in terms of internal reliability, construct validity, and ease of applicability:

#### c.1.) Validity

Construct validity was assessed by examining the correlation between the score and other measures of impact of the disease: modified Wood-Downes Score [WDS] (as the most widely used score in our setting) [9], length of stay, PICU internment, treatments received and duration of admission. Optimal cut-points methods were used to establish a severity threshold for the total score [10]. Pearson’s and Spearman’s Rho correlation coefficients were used to analyze correlation with quantitative variables. In order to compare the distribution of the score among groups of patients, Wilcoxon test and Kruskal-Wallis test were employed.

#### c.2) Reliability

Inter-observer reliability was assessed for the total score using a weighed kappa for each paired couple of investigators in G1, and for O1 and parents in the second cohort. For the first subset of patients, we also obtained the Light’s kappa for more than two observers (i.e, the mean of the kappa values obtained comparing each pair of investigators). Intra-class correlation coefficients were also used to assess inter-observer reliability. Internal consistency was evaluated using Cronbach’s alpha for each of the observers (O1, O2, and O3) and for the parental cohort. Scales with Cronbach’s alpha values >0.7 are considered to have good internal consistency [5, 11,12,13].

#### c.3) Utility

Individual items were reviewed for missing data and floor and/or ceiling effects. Percentages of missing data of less than 10% and floor/ceiling effects <15% are generally considered acceptable.

All statistical calculations were carried out using the packages *stats*, *psy* and *multilevel* from R Software, version 3.1.1. (https://www.r-project.org). Level of significance was set to *P*-value < 0.05.

## Results

**Fig 1:**
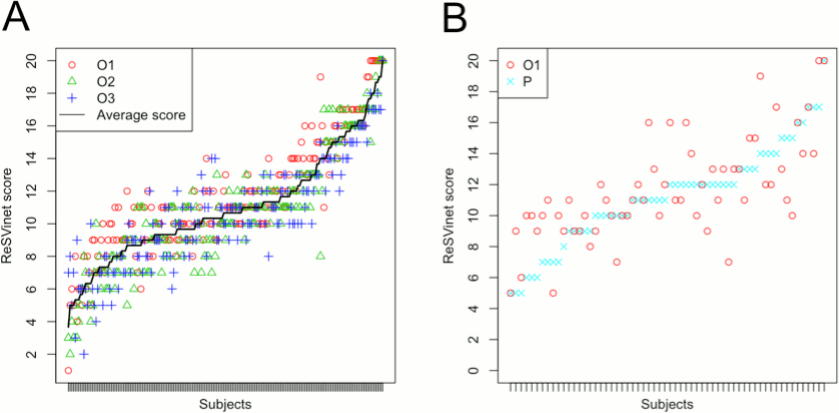
Comparison of results obtained (A) by the three observers, and (B) by the parents and one of the observers. In graph A: O1- Results obtained by observer 1. O2- Results obtained by observer 2. O3- Results obtained by observer 3. In graph B: O1- Results obtained by observer 1. P—Results obtained by the parents.

The three investigators completed all of the scales, whereas 60 of the 61 parents who agreed to do so completed all the items of the score (one of the patients was discharged without having his parents filled in their version of the score). Demographics and clinical aspects of “professional” and “parental” cohorts are summarized in **Tables 3 and 4**, respectively. Median age (interquartilic range) was 6.5 (2.0–9.5) months for the “professional” cohort while it was 2.3 (1.8–6.3) months for the “parental” cohort. Score results for each subject can be seen in **Fig 1**.

**Table 3:** Results of the ReSVinet scale in the professional cohort (Group 1). This table categorizes patients according to clinical, epidemiological and outcome variables in the cohort evaluated by three pediatricians.

			Observer 1	Observer 2	Observer 3	Least significant association
		Descriptive análisis^1^	Mean (SD)^2^	Mean (SD)^2^	Mean (SD)^2^	*P*-value^3^
ReSVinet Score			11.4 (3.4)	10.6 (3.5)	10.4 (3.1)	
Sex						0.101 (O3)
	Male	63.5% (108/170)	12.0 (3.4)	11 (3.6)	10.7 (3.1)	
	Female	36.5% (62/170)	10.5 (3.1)	9.8 (3.2)	9.8 (3.1)	
Age						0.790 (O2)
	< 1 year	85.3% (145/170)	10.9 (4.0)	10.3 (3.8)	10 (3.5)	
	1–2 years	14.7% (25/170)	11.5 (3.3)	10.6 (3.5)	10.5 (3.1)	
Previous wheezing episode						0.012 (O3)
	Yes	37.1% (62/167)	12.9 (3.5)	11.9 (3.9)	11.3 (3.6)	
	No	62.9% (105/167)	10.6 (3)	9.7 (3.1)	9.9 (2.7)	
Prematurity						0.928 (O3)
	Yes	8.4% (14/167)	12.6 (4.3)	10.7 (4.8)	10.7 (3.7)	
	No	91.6% (153/167)	11.3 (3.3)	10.5 (3.4)	10.4 (3.1)	
Nebulized epinephrine						0.120 (O3)
	Yes	57.6% (98/170)	12 (3.5)	11.1 (3.6)	10.7 (3.7)	
	No	42.4% (72/170)	10.7 (3.2)	9.8 (3.2)	10.4 (3.1)	
Antibiotics						< 0.001
	Yes	58.8% (100/170)	12.4 (3.5)	11.5 (3.4)	11.3 (2.9)	
	No	41.2% (70/170)	10.1 (2.6)	9.1 (3.2)	9.1 (3)	
Heliox						< 0.001
	Yes	37.3% (63/169)	14.1 (3.2)	13.3 (3.3)	12.4 (2.9)	
	No	62.7% (106/169)	9.9 (2.4)	8.9 (2.5)	9.2 (2.6)	
Suspected bacterian (super)infection						0.001 (O2-O3)
	Yes	41% (68/166)	12.6 (3)	11.6 (3.2)	11.5 (2.7)	
	No	59% (98/166)	10.7 (3.4)	9.9 (3.6)	9.7 (3.2)	
RSV detected^4^						0.739 (O2)
	Yes	77.1% (128/166)	11.4 (3.4)	10.6 (3.5)	10.7 (3)	
	No	22.9% (38/166)	11.7 (3.3)	10.6 (3.6)	9.8 (3.6)	
PICU admission						< 0.001
	Yes	21.8% (37/170)	15.7 (2.6)	15.4 (2.7)	14 (2.6)	
	No	78.2% (133/170)	10.2 (2.5)	9.2 (2.4)	9.4 (2.4)	
Wood-Downes Score						<0.001
	< = 3	27.6% (42/152)	9.6 (2.9)	8.2 (2.5)	8.6 (2.8)	
	4–6	50% (76/152)	11.1 (2.7)	10.2 (2.7)	10.2 (2.5)	
	≥7	22.4% (34/152)	15.1 (3.1)	14.7 (3.0)	13.7 (2.9)	
Hospital Length of Stay^5^		7.9 (3.9)	0.49	0.48	0.60	<0.001

^1^ Descriptive analysis: Data are expressed either as % (n/total of patients for which this condition was recorded in medical history) or as mean (SD).

^2^ Mean (standard deviation) of ReSVinet scale according to each rater and the different variables. Correlation was assessed by Wilcoxon’s test for dichotomic variables, Kruskal-Wallis for discrete variables with more than two categories and Spearman’s correlation for continuous variables.

^3^ The p-value on the table represents the least significant one among all the observers (the case presenting the least significant value is indicated between brackets).

^4^ The method used for detecting RSV (Respiratory Syncytial Virus) in respiratory secretions of our patients was direct immunofluorescence.

^5^ Spearman’s correlation coefficient was used for the statistical analysis. Mean length of stay expressed as days (SD).

**Table 4:** Results of the ReSVinet scale in the parental cohort (group 2). This table reflects the results of the score obtained by a physician (observer 1) and parents and the relation of the values obtained with clinical, epidemiological and outcome variables.

			Observer 1	Parent	Less significant association
		Descriptive análisis^1^	Mean (SD)^2^	Mean (SD)^2^	*P*-value^3^
ReSVinet Score			11.7 (3.3)	11.2 (3.3)	
Sex					0.968 (P)
	Male	68.3% (41/60)	12 (3.5)	11.3 (3.4)	
	Female	31.7% (19/60)	10.8 (2.8)	11 (3.1)	
Age					0.880 (O1)
	< 1 year	88.3% (53/60)	11.3 (3.4)	12.1 (4)	
	1–2 years	11.7% (7/60)	11.7 (3.3)	11.1 (3.2)	
Previous wheezing episode					0.672 (P)
	Yes	35.6% (21/59)	11.3 (2.5)	11.1 (3.1)	
	No	64.4% (38/59)	11.9 (3.7)	11.3 (3.4)	
Prematurity					0.135 (O1)
	Yes	8.5% (5/59)	14.6 (5)	13.8 (4)	
	No	91.5% (54/59)	11.4 (3)	11 (3.2)	
Nebulized epinephrine					0.281 (P)
	Yes	74.6% (44/59)	12 (3.2)	11.8 (2.9)	
	No	91.5% (54/59)	11.4 (3)	11 (3.2)	
Antibiotics					0.132 (P)
	Yes	50.8% (30/59)	12.7 (3)	11.9 (3.2)	
	No	49.2% (29/59)	10.7 (3.3)	10.7 (3.2)	
Heliox					0.002 (P)
	Yes	34.5% (20/58)	14.2 (3.3)	13.2 (2.7)	
	No	65.5% (38/58)	10.4 (2.5)	10.3 (3.1)	
Suspected bacterian (super)infection					0.173 (P)
	Yes	39.7% (23/58)	12.5 (3)	12 (3.6)	
	No	60.3% (35/58)	11.2 (3.4)	10.8 (3.1)	
RSV detected^4^					0.251 (O1)
	Yes	81.0% (47/58)	12 (3.3)	11.6 (3.2)	
	No	19.0% (11/58)	10.6 (3.5)	10.1 (3.8)	
PICU admission					< 0.001
	Yes	13.8% (8/58)	17.4 (2.1)	15.9 (2.5)	
	No	86.2% (50/58)	10.8 (2.5)	10.6 (2.7)	
Wood-Downes Score					<0.001
	< = 3	26.5% (13/49)	10.0 (2.2)	8.5 (2.8)	
	4–6	57.1% (28/49)	11.4 (2.6)	11.7 (2)	
	> = 7	16.3% (8/49)	16.8 (3.1)	15.9 (2.5)	
Hospital Length of Stay^5^		7.6 (2.8)	0.35	0.33	0.027 (P)

^1^ Descriptive analysis: Data are expressed either as % (n/total of patients for which this condition was recorded in medical history) or as mean (SD).

^2^ Mean (standard deviation) of ReSVinet scale according to each rater and the different variables. Correlation was assessed by Wilcoxon’s test for dichotomic variables, Kruskal-Wallis for discrete variables with more than two categories and Spearman’s correlation for continuous variables.

^3^ The p-value seen on the table is that of the least significant value from any of the observers (between brackets the observer presenting that value is indicated).

^4^ The method used for detecting RSV (Respiratory Syncytial Virus) in respiratory secretions of our patients was direct immunofluorescence.

^5^ Spearman’s correlation coefficient was used for the statistical analysis. Mean length of stay expressed as days (SD).

Cronbach’s alpha for the results obtained by each of the observers and for parental subgroup was greater than 0.70 in all cases (*r*-value ranged from 0.72 to 0.79) for the professional cohort (G1), 0.73 for the parental cohort (G2).

Weighed kappa values for each paired couple of investigators was 0.77 for O1-O2, 0.71 for O1-O3 and 0.74 for O2-O3 in the first cohort. Light’s kappa for more than two observers was 0.74. The weighed kappa for the group evaluated by parents and O1 was 0.73. There was a strong correlation between the results obtained by investigators (*r*-value ranged from 0.76 to 0.83) and also between the results obtained by the parents and the investigators (r = 0.73).

O1 allocated 2.4% of the patients evaluated to the extreme endpoints of the scale (Total score 0 or 20). Rates for O2 and O3 were 0.59% and 0.0%, respectively. Only 1.7% of children reviewed by their parents were given an extreme value.

Changes in the score depending on several clinical conditions were sought. Only those patients who were admitted to a Paediatric Intensive Care Unit (p<0.001 for both cohorts) or those receiving Heliox (a fixed mixture of 30% oxygen and 70% helium) (*P*-value < 0.001 for G1 and *P*-value = 0.002 for G2) were found to have a statistically significant higher score in both cohorts. A previous episode of wheezing could be related to a more severe disease in the sample assessed by professionals (P-value = 0.012) but not in the parental cohort (P-value = 0.672). RSV was identified as the agent causing the disease in 77.1% (G1) and 81% (G2) of the cases, but could not be definitively linked to a more aggressive course of infection (i.e, achieving higher scores in the scale). No differences were found in the score when comparing younger patients to older ones, regardless of the person carrying out the assessment, but this observation lacked statistical significance (*P*-value = 0.790 for G1 and *P*-value = 0.880 for G2). Interventions such as nebulized epinephrine and inhaled or oral corticosteroids could not be related with significant changes in the final score. Children on antibiotics did receive a higher score value in the paediatrician-assessed cohort G1 (mean difference range: 2.2–2.4), and this difference could be attributable to endovenous prescriptions (*P*-value < 0.001), but not to oral ones (*P-*value = 0.980). Similarly, scores of children with suspected bacterial super-infection presented also a greater score than those discharged without that suspicion (mean difference range: 1.7–1.9, *P*-value = 0.001).

Severity was defined by admission to PICU. Those infants who required intensive care presented a significantly higher score than those admitted to wards (15.7 ± 2.6 *vs*. 10.2 ± 2.5 when evaluated by O1, 15.4 ± 2.7 *vs*. 9.2 ± 2.4 for O2, 14 ± 2.6 *vs*. 9.4 ± 2.4 for O3 in the professional cohort, *P*-value < 0.001 for all of these values). These results could be replicated in the parental subgroup (15.9 ± 2.5 for *vs*. 10.6 ± 2.7 for those children assessed by their parents, *P*-value < 0.001). Cut-points analysis shows that a “severe episode” can be defined when a child scores ≥ 14 points in our scale. If severity of the episode was defined instead by obtaining a value in the “severe” range of the WDS (face value), we also found that values ≥ 14 in the ReSVinet scale were associated with values of ≥ 7 in the WDS; this association was found to bear statistical significance when comparing these patients to those who presented a “moderate” severity (defined by obtaining a 4, 5 or 6 in the WDS): in the first cohort results were 15.1 ± 3.1 *vs*. 11.1 ± 2.7 for O1, 14.7 ± 3.0 *vs*. 10.2 ± 2.7 for O2, 13.7 ± 2.9 *vs*. 10.2 ± 2.5 for O3; and parental values (G2) were 15.9 ± 2.5 *vs*. 11.7 ± 2.0 (*P*-value < 0.001 for all of these observations). Mean values and standard deviation from O1 for patients in parental cohort (G2) were 16.8 ± 3.1 *vs*. 11.4 ± 2.6. In both cohorts, higher results meant a higher WDS (*P*-value < 0.001 for G1 and G2).

Length of stay had a significant positive correlation with the total value of the scale (r: 0.48–0.60 for G1, *P*-value < 0.001, and r: 0.33–0.35 for G2, *P*-value = 0.027).

Parents found the scale easy to understand, and they could complete it in less than two minutes. For investigators, all the information required in the scale was accessible through electronic clinical records. Investigators needed about five minutes to review the clinical record of a patient.

## Discussion

The ReSVinet scale fulfills most of the requirements of an acceptable scoring system (**Table 5**) [5, 12,13,14] with the added value of avoiding the need for in-person evaluation and allowing an adapted version that is useful for non-professional assessment of the patient status.

**Table 5:** Check-list of the characteristics of the ReSVinet scale according to the desired properties of a clinical scale validated for use in infants with acute respiratory infections^*^. The “considerations” column explains whether the ReSVinet scale meets the requirement. N.A. = not assessed.

Property	Assessment	Considerations for the ReSVinet scale
Validity		
**Face validity**	Yes	The ReSVinet scale complies with 4 out of 5 points. Respiratory or heart rate (Q4), work of breathing (Q3), wheezing/auscultatory findings (Q3), mental status (Q6).
**Content validity**	Yes	Development of the score, target population and item selection are described in this paper.
**Construct validity**	Partially	No current gold standard. Used WDS, PICU admission and length of stay as criteria of severity for correlating hypothesis. The ReSVinet scale complied with all of them.
**Criterion-concurrent validity**	Partially	No current gold standard. Could be compared to SaO2 or pulmonary function (not done in this study), or cyanosis (deemed not feasible for a parent-oriented scale, as it could be difficult to evaluate).
Reliability		
**Measurement error**	N.A.	Absolute measurement error, usually expressed as smallest detectable change (SDC) i.e. the smallest within-person change in score that can be interpreted as real change above measurement error. Not evaluated.
**Inter-observer reliability**	Yes	Weighed kappa > 0.70 in a sample including more than 50 patients.
**Intra-observer reliability**	N.A.	Neither the investigators nor the parents re-evaluated their children.
**Internal consistency**	Yes	Cronbach’s alpha > 0.70.
**Responsiveness**	Ongoing	Currently being tested. Not evaluated in this paper.
Utility		
**Suitability for use in children**	Yes	No invasive techniques are required in this scale.
**Age span**	Partially	Although the ReSVinet Scale was designed to cover the entire pediatric lifespan, in this study it was tested only in children younger than 2 years of age.
**Ease of scoring**	Yes	< 4 categories per item.
**Auscultation skills**	Yes	No auscultation skills needed.
**Floor or ceiling effect**	Yes	<15% of patients with the lowest or the highest possible score in at least 50 patients.
**Interpretability**	Yes	Mean scores and standard deviation were calculated for clinical relevant subgroups. Results should be reassessed for patients older than 2 years, seen in outpatient settings or with chronic debilitating pathologies.

* List of items adapted from *Bekhof J*, *Reimink R*, *Brand PL*. *Systematic review*: *insufficient validation of clinical scores for the assessment of acute dyspnoea in wheezing children*. *Paediatric Respiratory Reviews*. *2014;15(1)*:*98–112*.

Our scale showed a good internal consistence and an acceptable inter-observer reliability even when tested by non-medical personnel. Most of the literature concerning scale validation deals with information obtained by only two investigators [5, 15], so having an acceptable weighted kappa index for more than two investigators is a relevant asset [5]. Intra-observer reliability or test-retest procedures can be assumed to be good if the inter-observer reliability is acceptable [12]. Hypotheses chosen as outcomes for testing face validity were correct for the WDS and discriminating the severity of the illness, and length of hospital stay also correlated positively with higher values of the scale in both groups.

So far, none of the many scores used for assessing the degree of dyspnea in patients with acute respiratory infections has been sufficiently validated to allow clinically meaningful use in children [5]. Our scale performed positively in 9 of the 15 items required. To the best of our knowledge, there are only a few scales whose validation criteria have been tested so thoroughly, and even fewer meeting more quality criteria than the ReSVinet scale [5]. The exclusion of different parameters by an expert panel is debatable, as there is ample evidence that some of them (such as peripheral oxygen saturation) are associated with longer admission and invasive measures [7]. The main points of disagreement concerned the inclusion of oxygen saturation and heart frequency, as both of them are frequently used in other scoring systems [16]. In the end, both were intentionally left out of the scale. Oxygen saturation is not always available in an outpatient setting. When used, recent studies show that its value in the decision-making process when facing a child with bronchiolitis may have been overestimated, leading to a higher rate of admissions [17]; physicians tend to abuse this “objective” measurement even when many do not understand its basis, placing great value on a difference of 2%, which is the accuracy most manufacturers report for their machines [18]. Parents also rely greatly on peripheral oxygen saturation when their child is admitted [19], and they could be prone to underestimate the true severity of the disease if this particular parameter is good enough for them. This variable was definitively discarded as its inclusion in the ReSVinet scale would hamper its usability by non-medical personnel–given the need of a device and training in probe placement and data interpretation–as well as its applicability in an outpatient setting. The need of oxygen supplementation (the intervention normally undertaken in patients with low oxygen saturation) is in any case covered in Q2. The “heart rate” parameter is featured in the most widely used scoring system, WDS [9], but its cut-off (120 beats per minute) is poorly discriminative in children under two years as it can be secondary to fever, irritability, pain, excitement, bronchodilators, or even a physiological heart rate in youngest ones. Another scores have defined higher cut-off values for this parameter. In the score designed by Marlais et al., the heart rate was the best of the five items chosen as predictors for admission[16], but since this was a retrospective study, this parameter was not compared with the heart rate of children being discharged directly from the emergency department; furthermore a higher heart rate may in any case be related to a more aggressive course of bronchodilators prior to admission. Wheezing is another item frequently included in respiratory assessment scales [4,6,9], but it can be difficult to explain, medical material (stethoscope) could be required for its assessment, and case definition of bronchiolitis in several countries does not require wheezing for the diagnosis [20, 21]. We chose instead to address the “respiratory noise” in Q3, as this concept is more understandable by non-medical personnel. The ReSVinet scale covers feeding difficulties (Q1), which have been related in several studies with greater risk of admission and invasive medical interventions [22,23,24] but are neglected by many of the scores already in use in clinical trials [4,5,7]. What is more, some of the most widely used scales deal only with the respiratory impact of the disease, like the Respiratory Disease Assessment Instrument (RDAI) or the Respiratory Assessment Change Score (RACS), both of which are widely used but have shown poor to moderate construct validity [4]; they even neglect findings such as hypoventilation and episodes of apnea. This particular concern is also found in scales more directed to asthmatic patients, like the Pulmonary Score [25]. Last, general condition is another parameter of our clinical scoring system which is usually not included in most of the existing scales, despite the fact that most clinicians seek and value the parental opinion on how severely affected they think their offspring are.

Our results suggest that this score has a reliable performance when calculating the respiratory difficulty from clinical records, making in-person evaluation not necessary. This feature could be of extraordinary value for clinical trials, where frequent in-person assessment and close follow-up is usually required when a participant presents with an adverse event such as bronchiolitis. Moreover, this could also be an asset in everyday clinical practice, allowing the primary care physician to obtain an impression of the condition of the infant at the moment of discharge from the emergency department, or their previous condition in a center that has just referred the patient for interment in another institution. In cases where medical records are not available or are not sufficient to allow the correct use of this score by the physician, parents could evaluate their children with results that can be compared to those of a physician. To the best of our knowledge, there is no other scale which shares these two advantages with the ReSVinet scale.

Limitations of our study include the lack of responsiveness in our currently gathered data, that the score was tested only on hospitalized patients younger than two years of age, and the inexistence of a gold standard for the evaluation of children with acute lower respiratory infection. Most of our patients suffered from acute bronchiolitis which, although the most frequent ARI during infancy, may not represent the whole spectrum of respiratory infections in the first 2 years. A memory bias was a possible confounding factor for the parents who filled in the score (as they completed it at the moment of discharge with the sole help of what they remembered), whereas quality and accuracy of clinical records could influence the results of the observers. Another reservation about the results presented in this study is that the professionals reviewing the clinical records were experienced pediatricians. Further testing may be required to ensure investigators with a different profile conduct the evaluation of clinical records with comparable precision. It could also be argued that clinicians may also bias the results as medical interventions influence the final outcome of the scoring system. However, the opposite could be argued too, as many decisions of pediatricians could be affected by the final score of any scale they use. Despite this potential risk of doctors’ decisions impacting the outcome of the scale, it was not possible to demonstrate that use of drugs, such as nebulized epinephrine or oral medication (corticosteroids or antibiotics), was associated with a significant increase in the final score in any of the two cohorts. In fact, severity range in the ReSVinet Scale correlated well with the definition of severity in the WDS, where medical interventions are not considered. It must be noted that there was a significant overlap between values that would correspond to “mild” and “moderate” episodes in the WDS, a finding that could be explained because the scale has only been applied to hospitalized children.

The final score value for patients in our setting did not show any difference dependent on the pathogen infecting them. In studies concerning other scoring systems, patients suffering from RSV had a higher total score [26]. Our scale failed to demonstrate any statistically significant impact of prematurity in the score, even though those children are at risk of worse episodes of bronchiolitis and many of them should receive prophylaxis against RSV [27]. Children of younger age in our study had a similar mean score to that obtained in older patients, although this finding lacked statistical significance. This can be explained by an insufficient number of subjects in both cohorts or the fact that we excluded children with chronic pulmonary conditions (which is a frequent problem in prematurely born babies); however, this outcome has already been described in medical literature [26], probably explained by the widely accepted measure of preemptively interning prematurely born children and younger infants. Aspects such as distance to a health provider, parental stress, or need for endovenous rehydration influence the decision of discharging a patient from the Emergency Department, and they are usually not gathered in respiratory scales nor clinical records. At the same time, a previous episode of wheezing was linked to a more severe disease in the cohort assessed by professionals but not in the parental cohort, which may be attributed to the significant lower mean age of the latter group. Neither the impact of maternal smoking during pregnancy nor breastfeeding were addressed in this study, although there is ample evidence of their influence in the severity of the disease [2].

The next steps for completing the validation of this score should include the application of this scale in outpatient settings, expansion of the age span of the scale to those children older than two years, and the inclusion of other pathologies which primarily manifest with respiratory difficulty in older children (asthma, atypical causes of pneumonia…) or in patients with underlying conditions. More importantly, the responsiveness of the ReSVinet scale should also be assessed. If the ReSVinet scale’s performance regarding responsiveness is acceptable, this scale will prove to be an important tool for the evaluation of infants suffering from respiratory infections.

## Conclusions

Our results indicate that the ReSVinet severity score has adequate criterion validity, adequate construct validity, adequate inter-rater reliability, and appropriate usability for infants under two years of age hospitalized for acute respiratory infections, even when used by non medical-personnel or when evaluating patients using clinical records and therefore avoiding the need for in-person evaluation of the subject by a physician. Although this scale still does not meet all the criteria for a perfectly validated scale, it complies with far more of them than the vast majority of the alternatives currently available and used in the clinical practice.

## Abbreviations

ARI: Acute Respiratory Infection
G1: Group 1
G2: Group 2
LOS: Length of Hospital Stance
O1: Observer 1 (Investigator 1)
O2: Observer 2 (Investigator 2)
O3: Observer 3 (Investigator 3)
P: Parents
PICU: Pediatric Intensive Care Unit
r: range
RDAI: Respiratory Disease Assessment Instrument
RACS: Respiratory Assessment Change Score
RSV: Respiratoy Syncitial Virus
WDS: Wood Downes Score
